# PP35 Keeping It Real Around The World: Comparing Real-World Evidence Guidance From Regulatory And Health Technology Assessment Bodies

**DOI:** 10.1017/S0266462324002071

**Published:** 2025-01-07

**Authors:** Yan Ran Wee, Alvin Ng, Eesha Dinkar, Jennifer Sara Evans

## Abstract

**Introduction:**

The growing use of real-world evidence (RWE) in pharmaceutical decision-making has prompted various guidelines, including REALISE (REAL World Data In ASia for HEalth Technology Assessment in Reimbursement). We compared RWE guidance from Asian, European, and North American health technology assessment (HTA) and regulatory bodies against the REALISE guidance.

**Methods:**

Following a previous search for China/Japan guidelines in 2022, websites of the U.S. Food and Drug Administration (FDA), the National Institute for Health and Care Excellence (NICE), the European Medicines Agency (EMA), and the Canadian Agency for Drugs and Technologies in Health (CADTH) were searched for RWE guidance in May 2023. Sections from each guidance were mapped onto REALISE and categorized as “agree,” “mixed,” “disagree,” or “missing” based on coverage/consistency.

**Results:**

Eleven guidelines were identified: four from Japan, three from China, and one each from FDA, NICE, EMA, and CADTH. No disagreements were found (all mapped sections were tagged “agree”/”mixed”); divergences were in coverage only. Most regulatory guidance had narrower scopes: EMA covered registry-based studies, the FDA’s framework mainly referenced other documents, while Japan had guidance for database and registry data. Conversely, HTA guidelines (CADTH, NICE) were more comprehensive and provided specific recommendations on preferred methods (e.g., transparent reporting) that were “missing” (45 to 46%) from REALISE’s more conceptual discussions. Chinese regulatory guidance was the exception, with similar coverage as REALISE (77% “agree”/“mixed”).

**Conclusions:**

Guidelines varied in scope, but there was overall concordance where recommendations could be mapped across documents. While regulatory bodies could focus on specific types of RWE, reflecting the specific role of RWE in regulatory evaluations (for example demonstrating safety), guidance for HTA was broader to account for different possible use cases in demonstrating comparative effectiveness and value.

